# Spermidine supplementation and protein restriction protect from organismal and brain aging independently

**DOI:** 10.18632/aging.206267

**Published:** 2025-06-07

**Authors:** YongTian Liang, Anja Krivograd, Sebastian J. Hofer, Laxmikanth Kollipara, Thomas Züllig, Albert Sickmann, Tobias Eisenberg, Stephan J. Sigrist

**Affiliations:** 1 Institute for Biology/Genetics, Freie Universität Berlin, Berlin 14195, Germany; 2 NeuroCure Cluster of Excellence, Charité Universitätmedizin Berlin, Berlin 10117, Germany; 3 Leibniz-German Center for Neurodegenerative Diseases (DZNE) Berlin 10117, Germany; 4 Institute of Molecular Biosciences, NAWI Graz, University of Graz, Graz 8010, Styria, Austria; 5 BioTechMed Graz, Graz 8010, Styria, Austria; 6 Field of Excellence BioHealth, University of Graz, Graz 8010, Styria, Austria; 7 Max Delbrück Center for Molecular Medicine (MDC), Berlin 13125, Germany; 8 Leibniz-Institut Für Analytische Wissenschaften – ISAS – e.V., Dortmund 44139, Germany; 9 Department of Chemistry, College of Physical Sciences, University of Aberdeen, Aberdeen, AB24 3FX, UK; 10 Medizinische Fakultät, Medizinische Proteom-Center (MPC), Ruhr-Universität Bochum, Bochum 44801, Germany

**Keywords:** aging, brain aging, spermidine, protein restriction, mitochondria

## Abstract

Brain aging and cognitive decline are significant biomedical and societal concerns. Both dietary restriction, such as limiting protein intake, and fasting, which restricts the timing of food consumption, have been proposed as strategies to delay aspects of aging. Recent studies suggest that intermittent fasting effects are mediated by the endogenous polyamine spermidine. Spermidine supplementation promotes mitochondrial integrity and functionality in aging brains by supporting hypusination of the translational initiation factor eIF5A. However, how molecular mechanisms underlying fasting mimicking interventions and protein restriction converge remain unclear, yet biomedically relevant.

In this study, we combined low- and high-protein diets (2% versus 12% yeast in food) with spermidine supplementation in aging *Drosophila* fruit flies. Effective hypusination was essential for normal life expectancy on both 2% and 12% yeast diets. Spermidine supplementation increased longevity, protected against age-related locomotion decline on both diets and improved memory scores in older flies regardless of protein intake. Notably, spermidine did not reduce the positive effects of the 12% protein diet on fecundity.

Our findings suggest that while both protein restriction and spermidine supplementation improve brain mitochondrial function, they largely operate through distinct mechanisms in modulating *Drosophila* brain aging. These results offer a basis for potential synergistic lifestyle interventions targeting age-related brain decline.

## INTRODUCTION

Dietary and lifestyle changes have been proposed as potential protective measures to delay the onset of organismal and brain aging [1–5]. Among these, dietary restriction (DR), including a reduction in the protein content from *ad libitum* food access, and fasting, which restricts the time window of food intake, or its mimetics, have garnered significant attention [5–10]. It is largely elusive whether these health-promoting interventions share a common mechanistic basis.

Spermidine (Spd) levels dwindle with aging in many tissues including the human brain [11–14]. Dietary Spd supplementation (Spd-S) was reported to extend the life- and healthspan across various model systems and animals [15, 16] Concerning brain aging, Spd-S has been previously shown to protect aging fruit flies and aging mice from mitochondrial dysfunction and cognitive decline [11, 16–21]. On molecular-mechanistic grounds, a specific post-translational modification called hypusination has been found to mediate Spd-S effects on mitochondrial integrity/functionality [18, 22] via a two-step enzymatic reaction involving deoxyhypusine synthase (DHS) and deoxyhypusine hydroxylase (DOHH) that occurs on the translational initiation factor eIF5A, where Spd serves as an amino-butyl donor [23, 24]. Spd-mediated extension and improvement in lifespan and locomotion are dependent on functional hypusination [18]. Spd-S promoted olfactory aversive learning, which relies on autophagy and mitophagy [11, 19, 22]. Most recently, elevations of the body-endogenous polyamine Spd have been proposed to mediate fasting effects from yeast over *Drosophila* to human cells, while blocking the Spd synthesis pathway blunted molecular effects and health benefits of fasting [25].

Spd-S alleviates mitochondrial dysfunction, cognitive decline, immune deficiency, and also other aspects of aging [11, 13, 16, 18, 26, 27]. Importantly, recent work showed that Spd levels increase upon fasting in yeast, flies, mice, human cells, and plasma from fasting volunteers [25]. As protein restriction (PR) is characterized by elevated brain mitochondrial abundance and functionality, increased longevity and protection against locomotion decay, PR might also display organismal Spd level changes.

Mitochondria are widely reported to undergo a functional decline with brain aging, a process meant to crucially contribute to functional brain aging [28–30]. Preserving brain mitochondrial integrity and metabolism with age is considered as a crucial factor in maintaining healthy brain function [31, 32]. Strategies aimed at enhancing mitochondrial function by targeting mitochondrial metabolism and quality control pathways hold promise for promoting healthy aging, protecting against age-related brain diseases, and ultimately increasing healthspan [33]. Indeed, neuronal signaling pathways activated by exercise and caloric restriction can stimulate mitochondrial biogenesis in the brain [34].

The relationship between DR and other fasting-mimicking strategies in organismal and brain aging remains a central question. To address this, we set out to combine DR, specifically, protein restriction (PR), with the fasting-mimicking intervention spermidine supplementation (Spd-S) in *Drosophila melanogaster*. We deliberately examined a broad spectrum of brain and organismal aging readouts, including age-sensitive behaviors such as locomotion and olfactory memory, alongside mitochondrial respiration and longevity measurements.

In this study, we observed that Spd-S elevated spermidine levels under both high-protein (12% yeast) and low-protein (2% yeast) conditions, although 2% yeast rearing alone resulted in low basal spermidine content. Low protein rearing boosted brain mitochondrial mass and respiration rates and partially protected against age-related locomotor decline, independently of hypusination pathways. Spd-S extended lifespan and ameliorated locomotor decline under both dietary conditions. Furthermore, although memory formation declined with age in flies on both diets, Spd-S robustly restored memory performance.

Our findings suggest that PR and Spd-S promote healthy aging through distinct and complementary mechanisms. Combining these interventions may offer enhanced benefits, providing valuable insights into potential dietary and pharmacological strategies to mitigate age-related cognitive and physiological decline.

## RESULTS

### Protein restriction, similarly to spermidine supplementation, promotes mitochondrial respiration in aging fly brains

Low protein diet in *Drosophila,* as in other organisms, refers to a reduction in food intake without malnutrition. We implemented established protocols by defining yeast extract concentrations as the key protein source in the fly food used [35, 36]. Dietary yeast content has previously been shown to regulate *Drosophila* lifespan [35]. We started by titrating food protein contents (1%, 2%, 5%, and 12% yeast w/v) to determine the dietary yeast concentration that would result in maximal lifespan under our conditions. For consistency reasons, we focused our study nearly exclusively on female flies [11, 18, 27]. As expected by previous reports [35], flies reared on a 2% diet lived the longest, while the 12% diet substantially reduced their lifespan (Supplementary Figure 1A). In quantitative terms, 2% rearing extended lifespan by 25% as compared to 12% rearing in female flies. Thus, we decided to concentrate on the comparison of flies reared in their adulthood (means post hatching) under 2% yeast (hereafter 2% flies) from flies reared on 12% yeast (hereafter 12% flies) for our analysis.

**Figure 1:**
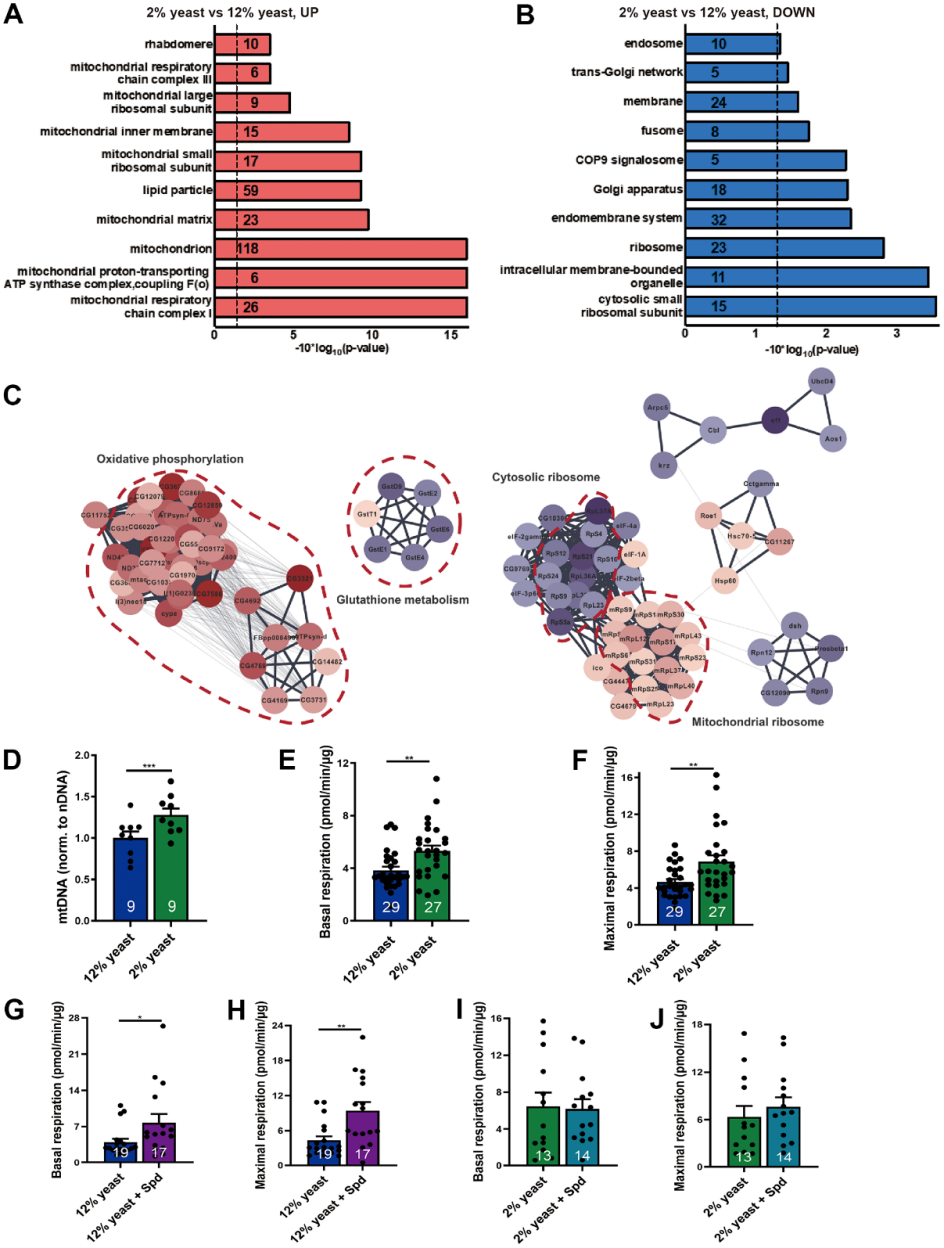
**Protein restriction increases mitochondrial mass, mitochondrial DNA copy number and promotes mitochondrial respiration in aging fly brains.** (**A**, **B**) Gene ontology analysis of iTRAQ global proteomics. Upregulated (red) and downregulated (blue) protein semantic groups in 15-day-old 12% yeast- and 2% yeast fed fly brains. Numbers indicate the numbers of proteins found in the individual semantic groups. (**C**) PPI network clustering analysis for the selected semantic groups of interest. Diagrams were created using DAVID software (version Dec. 2021). False discovery rate FDR p-values were reported here. (**D**) qPCR of mitochondrial DNA copy number relative to nuclear DNA in 15-day-old 12% yeast or 2% yeast fed fly brains (n= 9 biological samples. Each biological sample contains at least 3 fly brains). (**E**) Basal respiration of 15-day-old 12% yeast or 2% yeast fed fly brains. (**F**) Maximal respiratory of 15-day-old 12% yeast or 2% yeast fed fly brains. (**G**) Basal respiration of 15-day-old 12% yeast or 12% yeast + Spd fed fly brains. (**H**) Maximal respiration of 15-day-old 12% yeast or 12% yeast + Spd fed fly brains. (**I**) Basal respiration of 15-day-old 2% yeast or 2% yeast + Spd fed fly brains. (**J**) Maximal respiration of 15-day-old 2% yeast or 2% yeast + Spd fed fly brains. *p < 0.05, **p < 0.01, ***p < 0.001, ****p < 0.0001. ns, not significant. Data are mean ± SEM. P-values were determined by two-tailed paired t-test (**D**), or by two-tailed Mann-Whitney U test (**E**–**H**), or two-tailed unpaired t-test (**I**, **J**).

Seeking to explore the mechanistic relation of PR and high-protein diet, we first performed a quantitative proteomics analysis (isobaric tag for relative and absolute quantitation (iTRAQ)) screen comparing extracts of manually dissected isogenized wild-type *Drosophila* (*w^1118^*) brains from early aging (15-day-old) 2% and 12% flies. We identified 2397 protein hits in the global proteome analysis, from which 241 proteins were upregulated in 2% flies as compared to 12% flies with a cut-off of 1.5, while 73 proteins were downregulated with a cut-off of 0.667. Using the functional annotation tool from the DAVID bioinformatics resources [37], we found that most of the 2% rearing upregulated proteins fall into the electron transport chain (ETC) of mitochondria, especially mitochondrial respiratory chain complex I and III (Figure 1A and Supplementary Table 1). Besides, the terms mitochondrial matrix, mitochondrial inner membrane, and lipid particle were also associated with proteins enriched in 2% versus 12% yeast-reared *Drosophila* brains (Figure 1A). The downregulated protein set was enriched for cytosolic ribosomal proteins (Figure 1B).

Subsequent protein network analysis showed that a big cluster of proteins involved in oxidative phosphorylation and mitochondrial ribosome increased, while a small cluster of proteins involved in glutathione metabolism decreased (Figure 1C). Our findings are consistent with the increase of various nuclear encoded electron transport chain and mitochondrial ribosomal protein mRNAs upon DR in a polysome profile of whole *Drosophila* [38].

Our findings suggest that protein restriction can boost mitochondrial function in *Drosophila* brains. Mitochondrial DNA copy numbers were significantly increased in the brains of 15-day-old flies reared on 2% yeast compared to those reared on 12% yeast (Figure 1D). To assess whether mitochondrial respiratory activity was also enhanced, we used a Seahorse XF analyzer [18]. We found that 15-day-old flies reared on 2% yeast exhibited higher basal and maximal respiration compared to those reared on 12% yeast (Figure 1E, 1F), consistent with the elevated mitochondrial mass observed under low-protein rearing. Thus, 2% yeast rearing improves mitochondrial function in aging *Drosophila* brains, similarly to the effects of Spd-S [18, 19].

Along these lines, we previously demonstrated that Spd-S modulates the brain proteome under a normal diet [18], promoting mitochondrial abundance in aging *Drosophila* and supporting enhanced respiratory capacity. Importantly, we did not observe any detrimental effects of Spd-S under standard dietary conditions [11, 18]. Shedding further light on this, evidence from clinical studies in human volunteers has shown that Spd-S is safe and does not cause adverse effects [39]. Nonetheless, to exclude the possibility of detrimental effects of Spd-S under 12% and 2% yeast conditions, we performed proteomic analyses on 15-day-old flies reared on both diets with and without Spd-S (Supplementary Table 2) and found no substantial changes (Supplementary Table 2). In this context, we found that Spd-S increased mitochondrial respiration under the high-protein diet conditions (Figure 1G, 1H), whereas no significant change was observed under the low-protein diet conditions (Figure 1I, 1J), potentially because respiration is already substantially elevated in flies reared on 2% yeast compared to 12% yeast. We, therefore, pursued our analysis of the interaction between PR and Spd-S with the expectation that these interventions might converge mechanistically and, thus, potentially exhibit reciprocal occlusion of their protective effects.

### Spermidine supplementation and protein restriction promote lifespan extension and locomotor preservation through orthogonal mechanisms

**Figure 2:**
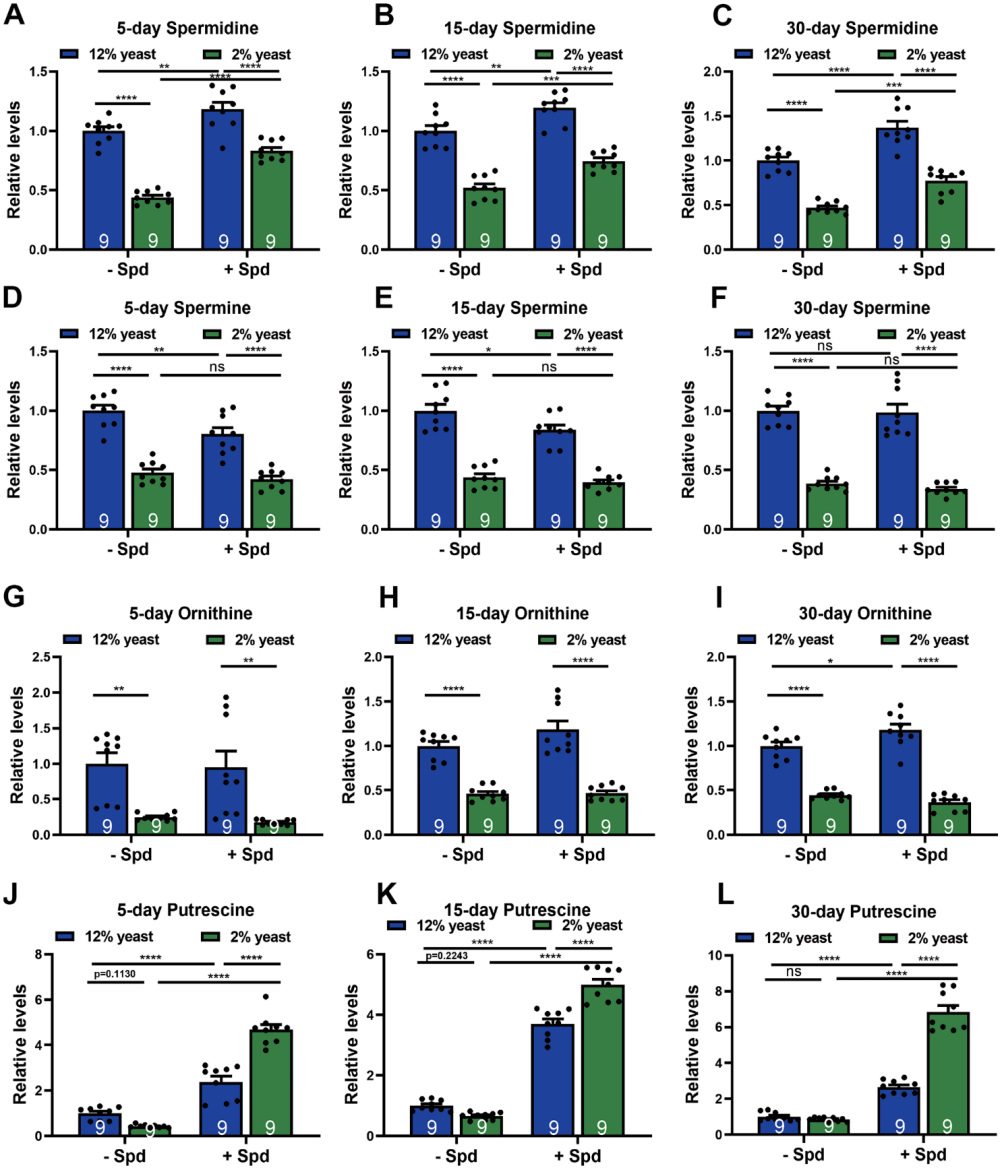
**Measurements of polyamine levels in wild-type *w^1118^* whole flies in different diets.** (**A**–**C**) HPLC-MS measurements of spermidine (**A**–**C**), spermine (**D**–**F**), ornithine (**G**–**I**) and putrescine (**J**–**L**) in wild-type *w^1118^* whole flies fed with 2% yeast, 12% yeast, 2% yeast + Spd, 12% yeast + Spd at 5-, 15- and 30-day-old time points respectively. n = 9 Biological samples. Each biological sample contains whole flies. Each biological sample has at least 10 whole flies for HPLC-MS measurements. *p < 0.05, **p < 0.01, ***p < 0.001, ****p< 0.0001, ns, not significant. Data are mean ± SEM. P-values were determined 2-way ANOVA with Tukey’s post hoc multiple comparisons test (**A**–**L**).

To monitor spermidine (Spd) levels in relation to the organismal effects on longevity associated with 2% versus 12% yeast rearing, and to validate the bioavailability of Spd-S across different protein conditions, we first assessed whole-organism Spd concentrations. We found that Spd levels decreased under 2% yeast rearing compared to 12% yeast rearing in age-matched groups (Figure 2A–2C). Other Spd precursors, such as spermine and ornithine, were also reduced in flies reared on 2% yeast (Figure 2D–2I). Given that ornithine is a direct product of arginine metabolism within the urea cycle, which converts excess ammonia generated from amino acid turnover [40], elevated Spd levels observed in 12% yeast-reared flies may reflect or result from increased protein and consequently amino acid intake.

As a prerequisite for testing the effects of Spd-S under conditions of protein restriction (PR), we also investigated whether dietary Spd-S would elevate Spd levels under PR, as low-protein conditions could potentially interfere with its uptake [11, 41, 42]. Importantly, organismal Spd levels were boosted in both 12% and 2% animals upon Spd-S (Figure 2A–2C), allowing us to further test for the effects of Spd-S in both conditions. In contrast to Spd, ornithine and spermine levels were essentially unchanged upon Spd-S (Figure 2D–2I). Notably, putrescine levels were markedly elevated upon Spd-S, drastically on the 2% diet (Figure 2J–2L). This could well be due to the metabolic conversion of dietary Spd-S into putrescine by the Spd degradation, as previously reported in *Drosophila* [11], or reflect a metabolic backlog of this Spd precursor as a result of decreased Spd synthase activity upon Spd-S. Taken together, 2% animals per se displayed lower Spd levels, which could be boosted upon Spd-S along with its precursor putrescine, Since Spd-S was successful in increasing Spd levels under different protein conditions, we decided to study the interplay of PR and Spd-S upon aspects of organismal and brain aging.

**Figure 3:**
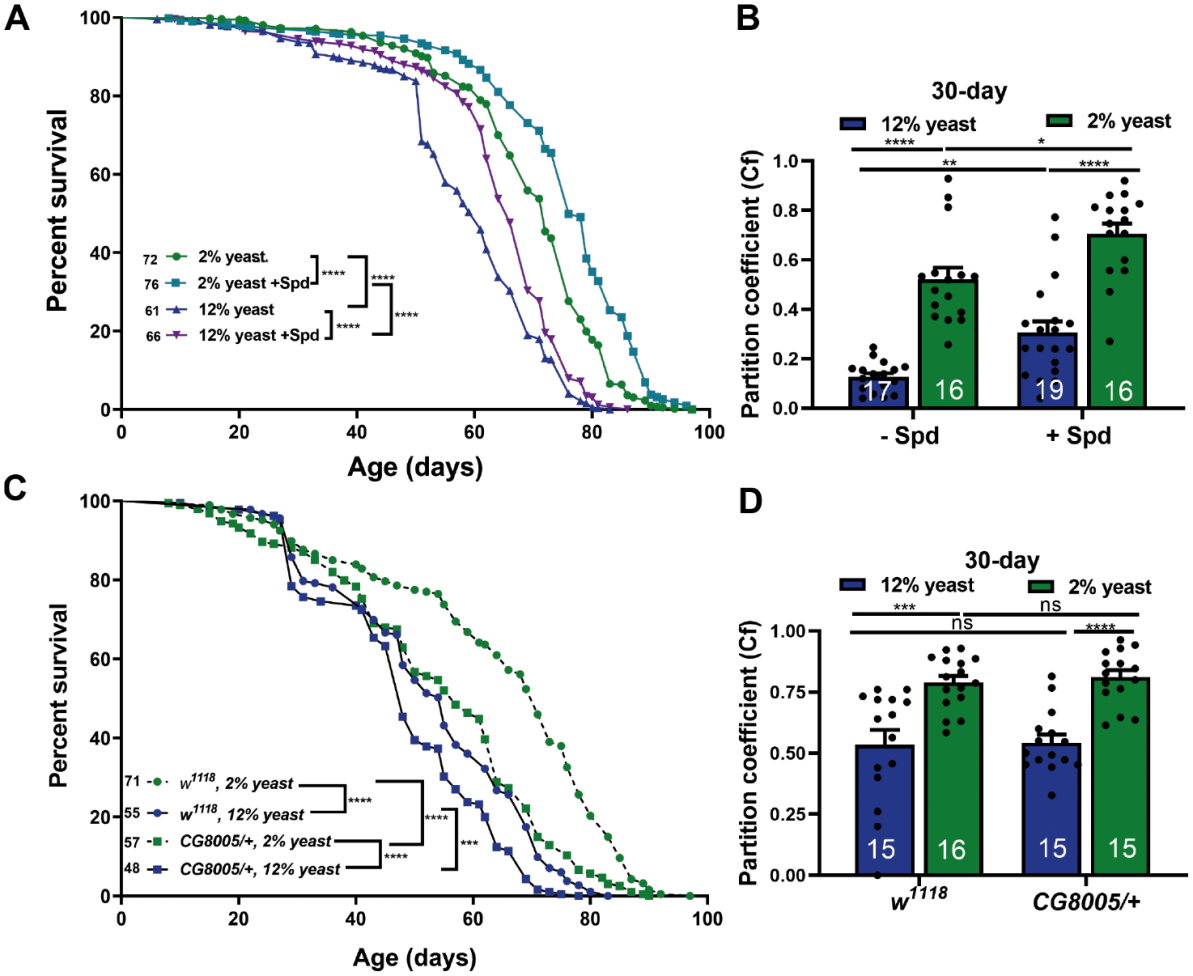
**Spermidine increases lifespan and ameliorates age-related locomotive decline in both 2% and 12% diets independent of hypusination.** (**A**) Survival analysis of isogenic *w^1118^* female flies, fed on 12% yeast and 2% yeast with and without addition of 5 mM spermidine. (**B**) Negative geotaxis of 30 days isogenic *w^1118^* female flies bred on either 12% or 2% with or without addition of 5 mM spermidine (n = 16 – 19 biological replicates. Each biological replicate contains around 15 female flies). (**C**) Survival analysis of isogenic *w^1118^* female flies and *CG8005/+* animals, fed on 12% yeast and 2% yeast. (**D**) Negative geotaxis of 30-day old isogenic *w^1118^* flies and *CG8005/+*, bred on 12% yeast or 2% yeast (n = 15 – 16 biological replicates. Each biological replicate contains around 15 female flies). *p < 0.05, **p < 0.01, ***p < 0.001, ****p < 0.0001. ns, not significant. Data are mean ± SEM. P-values were determined by the log rank test (**A**, **C**) and 2-way ANOVA with Tukey’s post multiple comparisons hoc test (**B**, **D**).

To explore the interplay of PR and Spd-S for readouts of organismal aging, we first measured the lifespan of flies combining Spd-S with the different yeast diets (Figure 3A). Interestingly, we here found that Spd-S significantly promoted longevity in both the 12% and 2% groups (Figure 3A). As a further measure of organismal aging, we analyzed locomotive abilities, previously shown to decay in aging fruit flies [43, 44]. Locomotor performance was assessed using a negative geotaxis assay, and the partition coefficient was calculated as a measure of locomotor activity [45]. Notably, minor variations in negative geotaxis assay conditions might affect the exact kinetics of divergence across experimental cohorts. Indeed, we demonstrated an age-related decline in locomotor activity in both 12% and 2% yeast diets and found that 2% rearing drastically ameliorated the age-related locomotive decline when compared to 12% rearing (Supplementary Figure 1B). Subsequently, we investigated whether Spd-S also protected locomotive abilities under these conditions. At 15 days of age, Spd-S already tended to preserve locomotive abilities in aging flies reared with 12% yeast (Supplementary Figure 1C). At 30 days of age, Spd-S executed significant protection of locomotion by Spd-S for both 12% and 2% rearing (Figure 3B). We conclude that Spd-S and PR execute protection on organismal aging of *Drosophila* in an additive manner. Thus, in regard to longevity, Spd-S and PR behaved additively and orthogonally. Consequently, hypusination, a spermidine-specific post-translational modification might still be important within 2% flies to arrive at maximal lifetime. To decisively rule out a functional role of hypusination in the PR-mediated age-protection effects, we utilized animals heterozygous for a null allele of *DHS/CG8005*, the rate-limiting enzyme for eIF5A hypusination [18, 46], a genetic constellation we previously found to be resistant to Spd-S effects [18].

We found that longevity in *CG8005/+* heterozygotes was significantly reduced under 12% yeast rearing, and even more markedly so under 2% yeast rearing (Figure 3C). Nevertheless, lifetime differences between 2% and 12% yeast diets persisted under hypusination-deficient conditions (Figure 3C). Moreover, 2% yeast rearing still significantly improved locomotion scores in *CG8005/+* animals, which displayed comparable locomotor abilities to *w^1118^* controls (Figure 3D). Therefore, PR-mediated lifespan extension is largely independent of hypusination.

Taken together, these findings indicate that Spd-S and PR mediate protection against organismal aging largely independently. We, therefore, proceeded to analyze how Spd-S and PR interact at the level of brain aging.

### Spermidine supplementation promotes memory formation independent of protein restriction in aging flies

Cognitive decline, particularly memory impairment, is common during normal brain aging [47–50]. Importantly, Spd-S has been shown to attenuate age-induced memory impairment in flies and mice [11, 18–20]. Our recent results further suggested that Spd-S mediated memory protection in aging *Drosophila* relies on functional hypusination [18].

**Figure 4:**
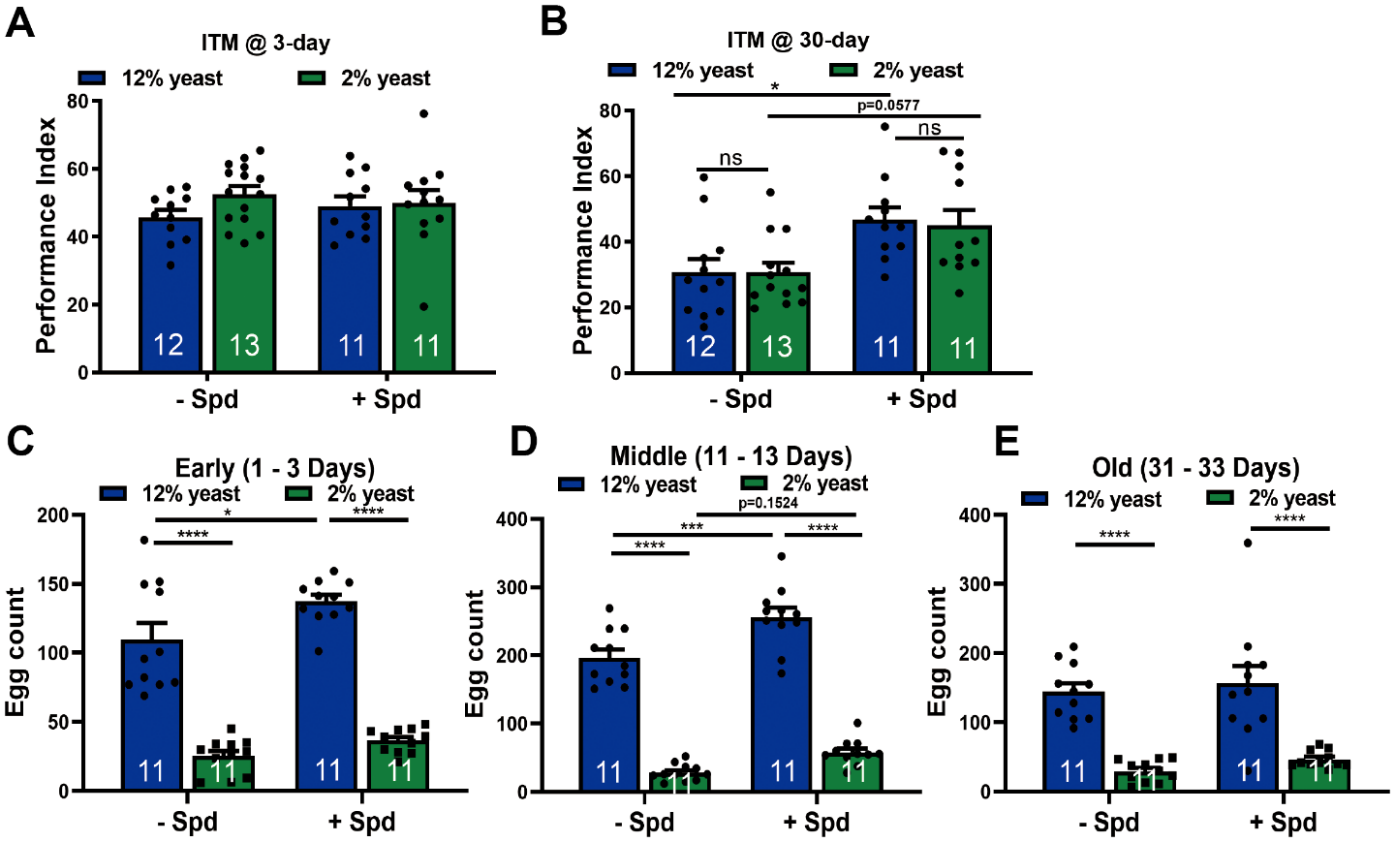
**Spermidine protects from memory amelioration in both 12% yeast and 2% yeast diets as well as boosts fecundity in 12% yeast in mid-age flies.** Intermediate-term memory (ITM) of 3-day (**A**) and 30-day (**B**) old isogenic *w^1118^* flies, bred on either 12% or 2% food with or without the addition of 5 mM spermidine (n = 11 – 13 biological replicates. Each biological replicate contains around 70 flies). (**C**–**E**) Fecundity assay of isogenic *w^1118^* female flies bred on 12% yeast and 2% yeast with and without the addition of 5 mM spermidine at early-, mid- and old-age respectively (n = 11 biological replicates). *p < 0.05, **p < 0.01, ***p < 0.001, ****p< 0.0001, ns, not significant. Data are mean ± SEM. P-values were determined by 2-way ANOVA with Tukey’s post hoc multiple comparisons test (**A** – **E**).

As a precondition of investigating memory function, we first tested whether principal olfaction (“smell scores”) would be affected in our experimental groups. No differences were detected, however (Supplementary Figure 2A, 2B). Thus, we investigated the relation between Spd-S and PR, specifically testing aversive olfactory intermediate-term memory (ITM), in young and aged animals (Figure 4A, 4B). In young animals, no differences in ITM scores were observed between the groups (Figure 4A), consistent with the finding that Spd-S does not promote memory formation in young but only aging animals [11, 18]. ITM scores dropped in both 12% but also 2% animals with aging, and no differences in memory performance were observed between 2% and 12% animals in age-matched groups (Figure 4A, 4B). Notably, however, by 30 days of age, Spd-S significantly enhanced ITM performance under both 2% and 12% rearing conditions (Figure 4B). Thus, Spd-S exerts age-sensible memory formation irrespective of PR. Interestingly, while *Drosophila* reared on 2% lived significantly longer, we did not detect improved ITM function at the timepoint analyzed.

### Spermidine supplementation further boosts fecundity under high-protein rearing

The disposable soma theory refers to an adaptive re-allocation of resources from reproduction to somatic maintenance [51–53]. When addressed from this perspective, lifespan extension upon PR and also Spd-S might be coupled with a reduction in female fecundity because both traits might be competing for limited resources [54]. To address this question, we measured egg laying in female flies across the early aging trajectory (up to 30 days of age). We observed significantly lower egg counts in flies reared on 2% yeast compared to 12% yeast at all assessed ages, irrespective of Spd-S treatment (Figure 4C–4E). During early and mid-age, Spd-S significantly stimulated egg laying under the 12% rearing condition (Figure 4C, 4D). Spd-S also tended to enhance egg laying in 2% low-protein-reared flies during mid-age (Figure 4D). Thus, despite its longevity-promoting effects, Spd-S did not impair fecundity in aging *Drosophila*.

## DISCUSSION

Understanding the connection between fasting-mimicking regimens, including DR and Spd-S, in the context of organismal and brain aging remains a central question. Given the practical challenges of directly combining these interventions, we adopted an alternative model by pairing protein restriction (a form of DR) with Spd-S in aging *Drosophila melanogaster*. Our analysis spanned a broad range of parameters, including mitochondrial respiration, longevity, and age-sensitive behaviors such as locomotion and olfactory memory.

In summary, our analysis revealed several key findings: (1) PR increased brain mitochondrial mass and respiration rates, and partially protected against age-related locomotion decline compared to a high-protein diet. Both readouts are similarly affected by Spd-S as we have shown previously [18]; (2) Spd-S elevated organismal Spd levels under both 12% and 2% protein conditions; (3) Spd-S extended lifespan and improved age-related locomotion across both dietary groups; (4) although memory formation declined with age under both protein conditions, Spd-S restored memory performance in both cases; (5) Spd-S further enhanced fecundity under the high-protein diet. These results suggest that Spd-S can “override” the protective effects of PR, providing additional benefits against organismal and brain aging, particularly in the context of a high-protein diet.

We found that PR increased mitochondrial mass and respiration in aging fly brains, while upregulating oxidative phosphorylation and mitochondrial ribosomal pathways and downregulating cytosolic ribosomal proteins. Although increased mitochondrial mass likely contributes to the higher respiratory capacity observed upon PR, further functional analyses of isolated mitochondria or application of mtDNA normalization strategies may help determine whether mitochondrial activity itself is also enhanced. Notably, however, these effects closely mirror the impact of Spd-S on *Drosophila* brains [18]. This observed shift is also consistent with previous findings that DR enhances the translation of nuclear-encoded electron transport components and mitochondrial ribosomal proteins in *Drosophila* [38]. From this perspective, PR might molecularly mimic Spd-S. This suggests that mitochondrial reprogramming alone may not fully account for the protective effects of Spd-S and PR, although differences in the magnitude of reprogramming could contribute to the distinct protective effects seen with 2% rearing versus Spd-S. Spd increases respiration rate even more, probably because exogenous Spd is uncoupled from the intracellular pool (which is lower in long-term 2% rearing).

Regarding the mechanism of protection, Spd shows cardioprotective effects in aging mice by reversing age-related mitochondrial structural decline. It improves mitochondrial organization, reduces size variation, increases mitochondrial number, and restores mitophagy and the balance between fusion and fission [17, 55]. It regulates mitochondrial quality via hypusination-dependent pathways, activating proteins like TFEB and ATG3 to promote biogenesis (via PGC1-α) and mitophagy (via PINK1-PRKN) [18, 22]. Functionally, Spd restores mitochondrial membrane potential, ATP production, and redox homeostasis while reducing ROS, with dose-dependent improvements in respiration observed in aged cells and neurons [56, 57]. Related polyamines like spermine similarly stabilize mitochondrial integrity and respiration, though through distinct mechanisms involving kinase-dependent and independent pathways [58]. Collectively, Spd and polyamines counteract aging-associated mitochondrial dysfunction via structural, biogenic, and functional enhancements, highlighting their multifaceted therapeutic potential.

The beneficial effects of Spd-S on lifespan extension and locomotion were found to depend on functional hypusination [18]. Notably, 2% protein rearing did not affect hypusine and eIF5A levels at a young age (Supplementary Figure 2C–2E). Brain-wide hypusine and eIF5A levels increased upon 12 % yeast feeding at 30 days of age compared to 2% yeast feeding (Supplementary Figure 2F, 2G), which is in line with the lower Spd levels found in this study. However, they remain similar between high- and low-protein feeding into older age (Supplementary Figure 2H–2K).

Importantly, PR extended lifespan in hypusination-deficient aging flies compared to 12% rearing. However, hypusination-deficient animals had significantly shorter lifespans under PR conditions, underscoring the importance of hypusination regardless of protein intake. Interestingly, Spd levels were higher in 12% reared animals than in PR-reared animals, likely due to the high amino acid content in their diet, indicating that hypusination of eIF5A is preferentially maintained and is decoupled from the low Spd levels under PR despite lower availability of polyamines. In this regard, Spd levels increased upon distinct regimens of fasting [25]. These findings and the discrepancy between PR and fasting concerning Spd levels demonstrated that the benefits of PR may arise from mechanisms other than Spd induction.

Caloric restriction as well as dietary protein restriction are known to regulate mTOR indirectly via AMPK activation and directly via various pathways, including insulin/IGF-1 signaling and differential abundance of amino acids sensed by TOR [59–61]. AMPK inhibits mTORC1 through Raptor phosphorylation, promoting autophagy [60, 62]. Phosphorylation of Raptor, which is a component of mTORC1 complex, and AMPK activation are key players in autophagy activation [62]. Spd is widely recognized for promoting autophagy, primarily through acetylation regulation and its impact on hypusination rather than direct mTOR inhibition. A review by Hofer et al. 2022 summarized the mechanisms of Spd-induced autophagy activation by acting as a direct substrate or a mediator [63]. Rarely, specific cases have shown mTOR inhibition by Spd [64] arguing for a rather cell- and context-dependent effect on mTOR by Spd, consistent with our finding that PR and Spd translate their health effects by largely independent mechanisms.

Despite the lack of demonstrable effects of PR on brain eIF5A hypusination levels, PR still promoted lifespan and locomotion in aging flies even under conditions of genetically challenged hypusination. Indeed, immunostainings demonstrated that hypusine levels were increased upon Spd-S under both 2% and 12% rearing conditions (Supplementary Figure 2L–2O). Other independent mechanisms, likely e.g. translation, might mediate the beneficial effects of Spd under PR and high-protein diet. However, regardless of the stage of regulation, mechanisms are clearly en route to boost mitochondrial proteins. Our findings largely exclude the possibility that PR exerts its anti-aging effects mechanistically via the spermidine-hypusination pathway. Instead, our results imply that PR might be mechanistically uncoupled from Spd-S effects. If so, both protective effects could potentially be additive or synergistic and mimic different molecular aspects of fasting, which would be of significant biomedical relevance. Along those lines, we found that PR did not improve ITM performance compared to a high-protein diet (Figure 4A, 4B). This outcome, however, aligns with previous research demonstrating that DR does not mitigate the age-related decline in aversive learning in *Drosophila* [35].

Combined interventions may interact to address different hallmarks of aging, potentially maximizing their effects on lifespan and healthy aging [65, 66]. In our study, we demonstrated that Spd-S combined with PR extended lifespan, improved locomotion and protected memory formation, outperforming PR alone. Notably, Spd-S also prolonged lifespan, enhanced locomotion, and preserved memory under a high-protein diet, showing that Spd-S can override PR effects. This finding should deepen our understanding of nutritional physiology in the context of combined interventions.

The trade-offs between reproduction and longevity have been controversial and might be sex-specific and context-dependent [54, 67–72]. Previously, Spd was suggested to have an evolutionarily conserved role in fertilization-related cellular fusion [73], and it has been found to rejuvenate oocyte quality by enhancing mitophagy during female reproductive aging [33]. The fact that Spd extends lifespan and promotes reproduction is worth investigating in greater detail. How Spd can decouple this trade-off might be of importance here. In short, nutritional supplementation with Spd under PR and high-protein diets appears to balance trade-offs between longevity, locomotion, memory, and reproduction (Table 1).

**Table 1:** A non-comprehensive summary comparing the differences and similarities between spermidine supplementation and protein restriction based on our study.

**Comparisons**	**Spermidine supplementation (Spd-S)**	**Protein restriction (PR)**
Spermidine levels	Increases spermidine levels at young age	Does not affect spermidine levels compared to the high protein diet
Hypusine levels	Increases hypusine levels at young age, in low- and high-protein diet as well as normal food backgrounds	Does not affect hypusine levels compared to the high protein diet
Longevity	Extends lifespan in low- and high-protein diet as well as normal food backgrounds	Extends lifespan compared to the high protein diet
Locomotion	Protects age-related locomotion decline at old age, in low- and high-protein diet as well as normal food backgrounds	Protects age-related locomotion decline compared to the high protein diet
Learning and memory	Protect from intermediate-term-memory decline at old age, in low- and high-protein diet as well as normal food backgrounds	Does not protect from intermediate-term memory decline compared to the high protein diet
mtDNA, mitochondrial respiration	Increases mtDNA and mitochondrial respiration	Increases mtDNA and mitochondrial respiration compared to the high protein diet
Fecundity	Boosts fecundity at young age in low- and high-protein diet as well as normal food backgrounds	Lowers fecundity compared to the high protein diet
Potential mechanisms of action	Enhances autophagy; promotes protein deacetylation; hypusination; anti-inflammation	Regulates mTOR signaling and insulin/IGF-1; amino acid metabolism

This study demonstrates the additive health benefits of PR and Spd-S through distinct, orthogonal mechanisms. However, important limitations must be acknowledged. The use of a single model organism, *Drosophila melanogaster*, restricts the broader applicability of our findings. While autophagy and hypusination pathways are highly conserved across species, replication in additional models with comparable functional readouts is essential.

We provided a non-comprehensive summary comparing the differences and similarities between Spd-S and PR based on our study (Table 1). To fully understand the anti-aging effects of various nutritional combinations, further mechanistic research is needed to elucidate how PR and Spd interact to influence mitochondrial function, proteomic profiles, and transcriptomic networks. It is also crucial to investigate the mechanisms by which Spd-S protects against age-related impairments across different contexts, including its potential when combined with other forms of DR or fasting. Although extrapolating to humans remains challenging, the strong conservation of aging-regulating mechanisms, anatomical parallels in certain organs, and the overlap in genes associated with aging diseases between flies and humans [74, 75] provide strong rationale for translation. We hope the findings presented here will motivate future complementary studies in mammalian systems to validate clinical relevance.

## MATERIALS AND METHODS

### Fly stocks and rearing conditions

All fly strains were reared under standard laboratory conditions, as previously reported [11], unless specially mentioned (25° C, around 70 % humidity with constant 12:12 h light/dark cycle). Flies from an isogenized *w^1118^* strain were used as the wild type control. Heterozygous *CG8005* mutants (*CG8005/+*) were originally obtained from Bloomington *Drosophila* Stock Center and outcrossed to *w^1118^* background for six generations. We manipulated adult diet by varying the yeast concentration; this is sufficient to manipulate longevity in flies [35]. The four diet recipes, with 10, 20, 50 or 120 g of dry inactive brewer’s yeast per liter of water, were referred to these diets as 1%, 2%, 5% and 12% diets, respectively. All diets contained 110 g of sucrose, 52 g of cornmeal, 7.9 g of agar, 2.3 g of methyl 4-hydroxybenzoate and 8.9 g of ethanol per liter of water [35]. Spd (Sigma Aldrich) was prepared as a 2 M stock solution in sterile distilled water, aliquoted in single-use portions and stored at −20° C. After the food had cooled down to 40° C, Spd was added to either 2% diet or 12% diet to a final concentration of 5 mM and was called ‘‘2% yeast + Spd’’ or ‘‘12% yeast + Spd’’. Parental flies mated on either 2% yeast diet or ‘‘2% yeast + Spd’’ food, and their progenies developed on the respective food for all experiments. Flies used in all experiments were F1 progenies. The flies in aging scenarios were collected within 24 h post eclosion and were, therefore, treated as 1-day-old flies. They were flipped to fresh food every other day until a specific age when they were subject to further processing.

### Extraction of 15-day *w^1118^* brains for iTRAQ global proteomics

Four samples (two biological replicates each from 2% yeast and 12% yeast groups) of dissected *Drosophila* brains (about 200 brains per condition) were homogenized in homogenization buffer containing 50 mM Tris (pH7.8), 150 mM NaCl and protease inhibitor cocktail, and further lysed in 4 % SDS. After lysis, brain lysates were centrifuged at 18,000 rcf at room temperature (RT) for 30 min. The clear supernatant was used for determining the protein concentration with a BCA assay, as per manufacturer’s instructions (Thermo Fisher Scientific, Germany). Aliquots corresponding to ~100 μg of protein of each sample were subjected to the reduction of disulfide bonds with 10 mM dithiothreitol (Roche, Germany) and incubation at 56° C for 30 min, followed by the alkylation of free thiol groups with 30 mM iodoacetamide (Sigma, Germany) and incubation at RT for 30 min in the dark. Sample cleaning and proteolysis (sequencing grade trypsin, Promega, USA) were performed using the filter aided sample preparation [76, 77] protocol with minor changes, as described previously [78]. Digested peptides were quality controlled [79] and the peptide concentration was determined using a NanoDrop 2000 UV-Vis spectrophotometer (Thermo Fisher Scientific, Germany). The samples were then dried in a SpeedVac and the dried peptides were resolubilized in 0.5 M triethylammonium bicarbonate buffer, pH 8.5 (Sigma, Germany). Afterwards, the labeling of peptides using iTRAQ (8-plex, AB SCIEX, Germany) was performed, according to the manufacturer’s instructions. The labeled samples were then multiplexed (pooled) and desalted with C18 SPEC tips (Agilent, Germany). Eluted peptides were dried in a SpeedVac and resolubilized in 10 mM NH_4_OH, 17 mM FA, pH 8.0. An amount of ~25 μg of multiplexed sample was fractionated by reversed phase chromatography at pH 8.0, as described earlier [78]. A total of 16 fractions were collected at 1 min intervals from min 5 to 80 in a concatenation mode. The fractions were completely dried and stored at -80° C until further use.

### LC-MS/MS analysis following isobaric tags for relative and absolute quantitation (iTRAQ) labeling

Each individual fraction was resolubilized in 30 μL of 0.1 % TFA and 50 % of the sample/fraction was analyzed using an Ultimate 3000 nano RSLC system coupled to a Q Exactive HF mass spectrometer (both Thermo Fisher Scientific, Germany). Peptides were preconcentrated on a 100 μm x 2 cm C18 trapping column for 10 min using 0.1 % TFA with a flow rate of 20 μL/min, followed by separation on a 75 μm x 50 cm C18 main column (both Acclaim Pepmap nanoviper, Thermo Fisher Scientific, Germany) with 90 or 120 min LC gradient ranging from 3–35 % of B (84 % ACN in 0.1 % FA) at a flow rate of 250 nL/min. The Q Exactive HF was operated in data-dependent acquisition mode and MS survey scans were acquired from m/z 300 to 1500 at a resolution of 60,000 using the polysiloxane ion at m/z 371.1012 as the lock mass [80]. The twenty most intense ions were isolated with a 0.4 m/z window and fragmented by higher energy collisional dissociation with a normalized collision energy of 33 %, taking into account a dynamic exclusion of 30 s. The MS/MS spectra were acquired at a resolution of 15,000. Automatic gain control target values and fill times were set to 3 × 10^6^ and 50 ms for MS and 2 × 10^5^ and 200 ms for MS/MS, respectively. Furthermore, a 10 % (v/v) NH_4_OH solution was placed in front of the ESI source for charge state reduction [81].

### iTRAQ data analysis and interpretation

All iTRAQ raw data were processed simultaneously using the MudPIT option with Proteome Discoverer 1.4 (Thermo Fisher Scientific, Germany) and searched against the *Drosophila melanogaster* proteome UniprotKB/TrEMBL (UP000000803) database with 22,009 target entries, downloaded on November 25, 2016, using Mascot (Matrix Science). Mass tolerances were set to 10 ppm and 0.02 Da for MS and MS/MS, respectively. Trypsin was selected as the enzyme with a maximum of two missed cleavages; carbamidomethylation of Cys (57.0214 Da) and iTRAQ-8-plex on the N-terminus and Lys (304.2053 Da) were set as fixed modifications, whereas the oxidation of Met (15.9949 Da) was a variable modification. Data export was done using the following filter criteria: peptide-spectrum matches with false discovery rate 1 % (high confidence setting), search engine rank 1 and only proteins that were quantified with ≥ 2 unique peptides were considered for further data analysis. Next, the normalization of the raw iTRAQ ratios was done using Excel (Microsoft), as described previously [78], to determine normalized abundance values (NAVs) for each protein. The respective replicates were averaged accordingly using these NAVs, and the ratios were calculated between different conditions, for example, 2% yeast / 12% yeast food. The ratio cutoffs for differential protein abundances were ≤ 0.667 for down- and ≥ 1.5 for upregulation, respectively.

### iTRAQ proteomics analysis

The functional annotation tool from DAVID bioinformatics resources was applied to analyze the GO (GO-term) [37]. The proteomics PPI network analysis was performed using Cytoscape [82] with STRING [83] plugin and MCODE [84] for the clustering analysis.

### Label-free quantification (LFQ) whole- fly global proteomics

Ten female adult fly bodies of a designated age were immobilized on ice and placed into a single Eppendorf tube. The samples were lysed in 100 μL of lysis buffer containing 50 mM Tris-Cl (pH 7.8), 150 Mm NaCl, 2% SDS plus Protease Inhibitor Cocktail (Roche #11836170001) using a mechanical homogenizer. To degrade nucleic acids, cell lysates were treated with Benzonase plus 2 mM MgCl_2_ and incubated at 37° C for 30 min. Next, cell lysates were centrifuged at 18,000 rcf at room temperature (RT) for 30 min. Clear supernatant was used for determining protein concentration with the BCA assay as per manufacturer’s instructions (Thermo Fisher Scientific, Germany). Lysates corresponding to 100 μg of protein of each sample were subjected to carbamidomethylation with 10 mM DTT and incubation at 56° C for 30 min (reduction of disulfide bonds) followed by the alkylation of free thiol groups with 30 mM IAA and incubation at RT for 30 min in the dark. Sample cleaning and proteolysis (trypsin) were performed using the S-Trap mini protocol (Hentschel A, Rasband W, Eliceiri K. S-Trap mini spin column digestion protocol. ProtiFi LLC. 2021. https://www.protifi.com). Next, the digests were completely dried in a SpeedVac, and the dried peptides were resolubilized in 0.1% TFA followed by evaluation of digestion efficiency on a Monolithic HPLC [79]. Each sample was adjusted/normalized based on the peak areas (Ultraviolet @ 214 nm) such that same number of peptides were analyzed by nano-LC-MS/MS using an Ultimate 3000 nano RSLC system (C18, 120 min gradient ranging from 3-30% of B) coupled to an Orbitrap Eclipse mass spectrometer (both Thermo Fisher Scientific, Germany) in data dependent acquisition mode.

Data Analysis was conducted with Proteome Discoverer (PD) 2.5 software using the precursor-based label-free quantitation (LFQ) workflow nodes. MS spectra were searched against the *Drosophila melanogaster* reference database (downloaded on 30.03.2021) using the Sequest HT algorithm. Trypsin with a maximum of two missed cleavages was selected as an enzyme. Carbamidomethylation of Cys was set as fixed, and oxidation of Met was selected as a variable modification. MS and MS/MS tolerances were set to 10 ppm and 0.5 Da, respectively. False discovery rate (FDR) validation on the peptide-spectrum match (PSM) level was done using the Percolator node. Peak and feature detection were done by the “Minora” feature detector node using default parameters. In the Consensus workflow of PD, the peptide and protein filters were set to an FDR of 1% and default settings of the “Feature Mapper” node were employed. For the “Precursor Ions Quantifier” node, unique peptides were set to use and “Precursor Abundance Based On” was set to intensity. “Normalization Mode” was set to the total peptide amount and for “Scaling” the normalized abundances; on all averages were selected. “Protein Abundance Calculation” was done by using the summed abundances and “Protein Ratio Calculation” was set to protein abundance based. For missing values, the “Imputation Mode” was set to low abundance resampling. UniProt. Only proteins quantified with ≥2 unique peptides, ≤1% FDR, and holding a quan value >0 were included in downstream analysis.

### Measurement of mitochondrial respiration using seahorse XFe96 analyzer

Adult fly brains of the appropriate genotype and age were dissected and collected in supplemented Schneider’s medium (Schneider’s *Drosophila* medium (Gibco), 10 % fetal bovine serum (Sigma), 2 % penicillin-streptomycin (Sigma)), as described previously [18, 85]. Following centrifugations, fly brains were washed twice in Rinaldini’s solution, and digested enzymatically in Rinaldini’s solution containing collagenase I (Sigma; 1 mg/mL) and papain (Sigma; 1 mg/mL) for 30–40 min at 25° C in a thermomixer [86]. Brain samples were washed once with Rinaldini’s solution and then washed twice with Schneider’s medium containing sodium pyruvate (Sigma; 1 mM). Brain tissues were pipetted repeatedly after digestion to dissociate brain cell clusters into cell suspension in Schneider’s medium containing sodium pyruvate. Cell solutions were filtered homogeneously through 40-μm cell strainers (Thermo Fisher Scientific, Germany) and cells were seeded in Cell-Tak-coated (BD Biosciences, NJ, USA) 96-cell culture microplates (Seahorse Bioscience, Agilent, Germany). The assay was performed in Schneider’s medium containing 1 mM sodium pyruvate, as described previously [85]. The hydration of a Seahorse XF96 sensor cartridge was performed one day prior to the assay. The loading of compounds (Oligomycin: complex V inhibitor; FCCP: proton gradient uncoupler; Antimycin A and Rotenone: complex I, III inhibitor) and calibration of the cartridge sensor were performed on the day of the assay. Following instrument calibrations, cells were transferred to the XFe96 analyzer to record the OCR at the appropriate temperature. After the assay, the medium was carefully aspirated. Cells were lysed with RIPA buffer, and the BCA assay kit was used to measure the total protein concentration to correct for protein levels.

### Mitochondrial DNA measurement

The total DNA from 30 adult brains (2% yeast and 12% yeast) were extracted in the NucleoSpin Tissue kit (Macherey-nagel GmbH, Germany), according to the instructions. A volume of 15 ng of total DNA from respective brain samples was used in quantitative PCR. Mitochondrial DNA was quantified relative to nuclear DNA by the ratio of amplicons of mitochondrial-encoded *cytochrome oxidase subunit I* (*COI*) to amplicons of nuclear-encoded *glyceraldehyde 3-phosphate dehydrogenase* (*GAPDH*) in quantitative real-time PCRs. Primer sequences were reported as follows [87]: COI, GAATTAGGACATCCTGGAGC and GCACTAATCAATTTCCAAATCC; GAPDH, GACGAAATCAAGGCTAAGGTCG and AATGGGTGTCGCTGAAGAAGTC.

### Polyamine measurement in whole flies

Extraction of polyamines from whole flies was performed as follows: 30-50 mg of frozen whole flies were homogenized in 1200 μl ice-cold 5% trichloroacetic acid (TCA) aqueous solution using an UltraTurrax device (IKA T10 basic) for 10-15 sec. 150 μl of the resulting homogenate diluted with 5% TCA to a final volume of 400 μl containing 100 ng/ml stable-isotope labeled polyamines as internal standards as described [88], followed by incubation on ice for 1 h with vortexing every 15 min. After centrifugation at 25,000 g, 4° C; 10 min, 150 μl of the supernatant were transferred to 1.5 ml Protein LoBind reaction tubes (Eppendorf, 0030108116) and subjected to polyamine derivatization by isobutyl chloroformate as described [88] using a modified carbonate buffer (1 M ammonium bicarbonate, pH 10) and followed by offline SPE purification as described in [89]. After elution with 250 μl 80% acetonitrile (containing 0.2% acetic acid), 5 μl of the eluate were subjected to LC-MS analysis. LC-MS method to analyze polyamines was an adapted version of Magnes et al. (2014) [88]. UHPLC 1290 Infinity II combined with a Triple Quadrupole (TQ) 6470 (both Agilent, Waldbronn, GER, Germany) with an Agilent Jet Stream ESI source was used for the analysis. The LC separation was performed on an EclipsePlus C18 column (2.1x50mm, 1.8μm, Agilent, Waldbronn, GER, Germany) with a gradient elution. Therefore, water-based eluent A and isopropanol-based eluent B with ammonium acetate (10 mM), phosphoric acid (8 μM) and formic acid (0.1 vol%) were used. The LC gradient was started with 25 % eluent B for 0.3 minutes, increased to 100 % and held for 0.4 minutes before being lowered to 25 % of the initial condition and recalibrated for 1.29 minutes at a flow rate of 0.4 ml/min. The TQ was used in positive and multi-reaction monitoring (MRM) mode. The ESI spray voltage was set to 3 kV, the sheath gas flow to 12 l/min at 350° C, the gas flow to 5 l/min at 260° C, and the nebulizer pressure was 30 psi. MRM transition are described in Magnes et al. (2014) [88], the following adjustments were made: DynamicMRM mode was used for the scan time, and the voltages for the collision energy (CE) and fragmentor (Frag) were used as follows: Orn (112 Frag, 21 CE), put (80 Frag, 13 CE), sp (208 Frag, 45 CE), spd (144 Frag, 13 CE), D_8_-put (144 Frag, 14 CE), ^13^C_5_-orn (112 Frag, 20 CE), ^13^C_4_-spd (144 Frag, 15 CE), and D_8_-sp (144 Frag, 45 CE). Data integration was done with Skyline (https://skyline.ms/skyline.url) and quantification was performed using weighted (1/x^2^) linear regression based on the ratio of analytes and internal standard and was finally normalized to the respective sample’s wet weight.

### Negative geotaxis measurements

Female and male flies of the right genotypes and ages were sorted at least two days before the assay. A chamber temperature around 24–25° C and humidity of 40–60 % was maintained before and during the assay, as described previously [45]. Ten test tubes were placed on the countercurrent apparatus. The flies were transferred into the eleventh tube using a funnel and the tube was placed to the lower far left. After 3 min of acclimation, the apparatus was held and banged down strongly five times. The upper frame was slid from the right to the left, switching between the ‘transfer state’ and ‘testing state,’ respectively. After five sessions, the number of flies in each tube was recorded and the partition coefficient Cf for each strain was calculated using the formula: Cf = (N_2_+2N_3_+3N_4_+4N_5_+5N_6_)/5(N_1_+N_2_+N_3_+N_4_+N_5_+N_6_).

### *Drosophila* lifespan experiments

Isogenized *w^1118^* strain was used in longevity experiments. Females and males were allowed to mate for 48 h before being sorted. A total of 100 3-day-old female flies were collected for each of the three biological replicates for each condition. A total of 20 *w^1118^* females were put in their respective food (1% yeast, 2% yeast, 5% yeast and 12% yeast food). Fresh food was changed and records of the number of dead flies were carried out three times a week. Flies that escaped during flipping were excluded from the survival censorship. Similarly, another pair of comparisons (*w^1118^* and *CG8005/+*) were also subjected to survival analysis. The comparison of survivorship data was performed using the log rank test.

### Olfactory aversive learning

Behavioral experiments were performed in dim red light around 25° C and 80 % relative humidity with 3-Oct (1:100 dilution in paraffin oil presented in a 14-mm cup) and MCH (1:100 dilution in paraffin oil presented in a 14-mm cup) serving as olfactory cues and 120V AC current serving as a behavioral reinforcer, as described previously [90]. Standard single-cycle olfactory associative memory was performed, as described previously, with minor modifications. Briefly, about 80–100 flies received one training session, during which they were exposed sequentially to one odor (conditioned stimulus, CS+; 3-Oct or MCH) paired with electric shock (unconditioned stimulus) and then to a second odor (CS−; MCH or 3-Oct) without unconditioned stimulus for 60 s, with a 60 s rest interval between each odor presentation. During the testing, flies were exposed simultaneously to the CS+ and CS− in a T-maze for 60 s. The conditioned odor avoidance was tested immediately after training for STM (memory tested immediately after odor conditioning). Subsequently, flies were trapped in either T-maze arm, subsequently anesthetized and counted. A performance index was calculated from this distribution as the number of flies avoiding the CS+ minus the number avoiding the CS− divided by the total number of flies and, finally, multiplied by 100. A 50:50 distribution (no learning) yielded a performance index of zero, and a 0:100 distribution away from the CS+ yielded a performance index of 100. A final performance index was calculated by the average of both reciprocal indices for the two odors. Flies were trained for MTM, as described above, but tested 3 h after training.

### Fecundity

Fecundity experiments were carried out similarly to the procedures described by Krittika and Yadav with minor adaptations [91]. Wild type flies were reared on either 2% yeast or 2% yeast supplemented with Spd, as previously designated. 1-day-old F1 generation flies were collected upon hatching. Pairs consisting of 10 females and 10 males were placed in vials under four different dietary conditions as follows: flies reared on 2% yeast were placed on 2% and 12% yeast respectively, and flies reared on 2% + Spd were placed on 2% + Spd and 12% + Spd food respectively. Egg counts commenced on day 1 and continued through day 33. During this period, egg counts were assessed over four developmental stages, each monitored for three consecutive days: early (days 1-3), mid (days 13-15), late (days 21-23), and old (days 31-33). Eggs were counted at 9 AM and 5 PM, with flies being transferred to fresh food at each count. More flies of the same condition were aged in parallel to always ensure a constant number of 10 pairs. Data were pooled from three independent experiments, with 11 data points per dietary condition, yielding an n of 110 female and male pairs. Egg counts were normalized to the counts from the 12% food group at each developmental stage.

### Whole-mount immunostaining, confocal imaging and quantification

Adult brains were dissected in Ringer’s solution on ice and immediately fixed in cold 4 % paraformaldehyde (w/v) for 30 min at RT. After fixation, samples were washed three times for 10 min each with 0.7 % PBT (phosphate-buffered saline (PBS) containing 0.7 % Triton X-100, v/v) and then blocked with 10 % normal goat serum in PBT (v/v) for 2 h at RT. After blocking, samples were incubated in 0.7 % PBT containing 5 % normal goat serum and the primary antibodies for 48 h at 4° C. After primary antibody incubation, brains were washed in 0.7 % PBT six times for 30 min each at RT and then incubated in 0.7 % PBT with 5 % normal goat serum containing the secondary antibodies overnight at 4° C. Brains were washed six times for 30 min each with PBT at RT and mounted in Vectashield (Vector Labs, CA, USA). The following antibodies and dilutions were used in whole-mount adult brain staining: rabbit anti-hypusine antibody (Merck; 1:2000), guinea pig anti-eIF5A antibody (customized; 1:200), goat anti-rabbit Cy5 (Invitrogen; 1:300). Image stacks of specimens were imaged on a Leica TCS SP8 confocal microscope (Leica Microsystems, Germany) using a 40×, 1.3 NA oil objective for whole-brain imaging with a voxel size of 0.3788 x 0.3788 x 0.9997 micron^3. Images were quantified using ImageJ software (https://fiji.sc/). Briefly, the average intensity z-projection was performed with ~ 100 stacks of each brain from the antennal lobe to the end of the antennal lobe and the mean grey value of the central brain was measured.

### Quantification and statistical analysis

All statistical analyses were performed using Prism software (GraphPad). All results are presented as mean ± SEM. Shapiro-Wilk normality tests were performed before choosing the statistical test. If a dataset was not normally distributed, nonparametric analyses were applied. Unless otherwise noted, statistics were based on two-tailed unpaired Student’s t-tests or Mann–Whitney *U* tests for two-group comparisons. One-way ANOVA was used, followed by Sidak’s *post hoc* analysis for the comparison of multiple groups unless otherwise stated. A non-matching two-way ANOVA was used, followed by Tukey’s *post hoc* analysis for the comparison of two or more groups across the treatment condition, genotype or time. A 2-tailed p < 0.05 was considered significant (*p < 0.05, **p < 0.01, ***p < 0.001, ****p < 0.0001). Our sample sizes were similar to those reported in previous publications [11, 27].

### Contact for reagent and resource sharing

Further information and requests for resources and reagents should be directed to and will be fulfilled by the Lead Contact, Stephan J. Sigrist (stephan.sigrist@fu-berlin.de).

### Data availability statement

The data that support the findings of this study are available on request from the corresponding author. The data are not publicly available due to privacy or ethical restrictions.

## Supplementary Material

Supplementary Figures

Supplementary Table 1

Supplementary Table 2
