# Supplementary material for: Spermidine supplementation and protein restriction protect from organismal and brain aging independently
Source: Aging (Albany NY). 2025 Jun 7;17(6):1429–51. doi: 10.18632/aging.206267 (PMC12245200; doi:10.18632/aging.206267)
Supplement: Supplementary Figures [file aging-17-206267-s001.pdf]

# SUPPLEMENTARY FIGURES

## Supplementary Figures

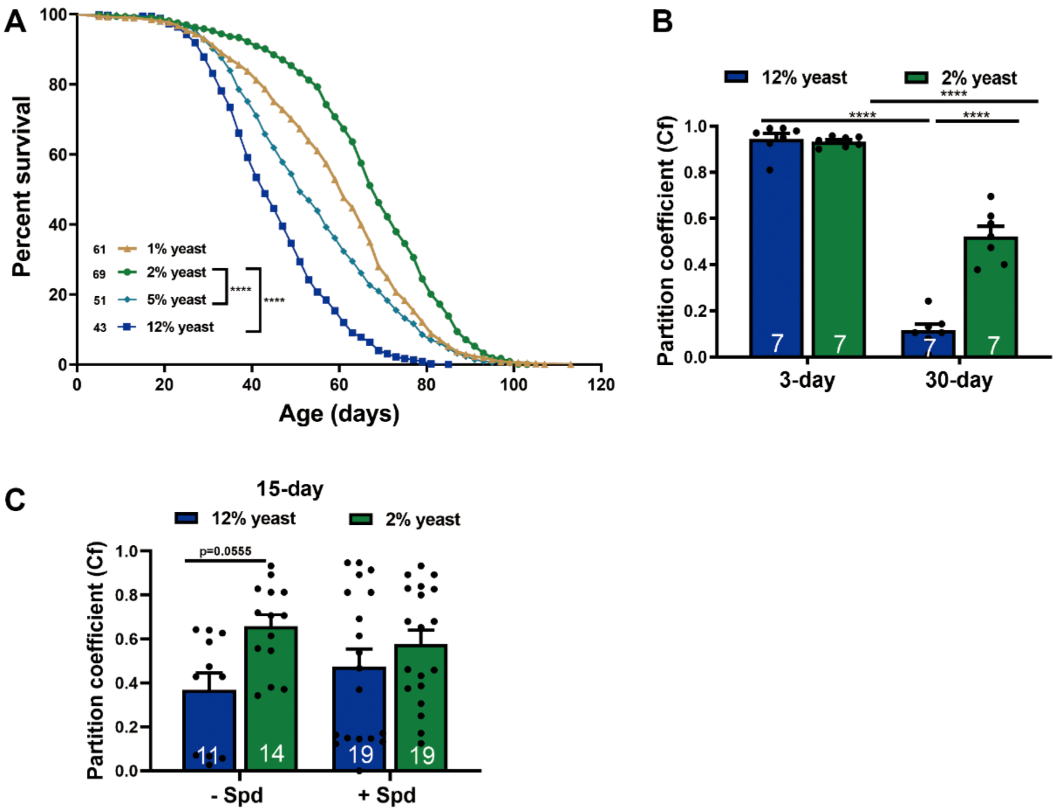


**Supplementary Figure 1. Protein restriction increases lifespan and ameliorates locomotive decline.** (**A**) Survival analysis of isogenic $w^{1118}$ female flies, fed on 12% yeast and 2% yeast. (**B**) Negative geotaxis of isogenic $w^{1118}$ female flies bred on 12% yeast or 2% yeast at 3- and 30-day (n = 7 biological replicates. Each biological replicate contains around 15 female flies). (**C**) Negative geotaxis of 15 days isogenic $w^{1118}$ female flies bred on 12% yeast and 2% yeast with and without addition of 5 mM spermidine (n = 11 – 19 biological replicates. Each biological replicate contains around 15 female flies). *p < 0.05, **p < 0.01, ***p < 0.001, ****p< 0.0001, ns, not significant. Data are mean ± SEM. P-values were determined by the log rank test (**A**) and 2-way ANOVA with Tukey's post hoc test (**B**, **C**).

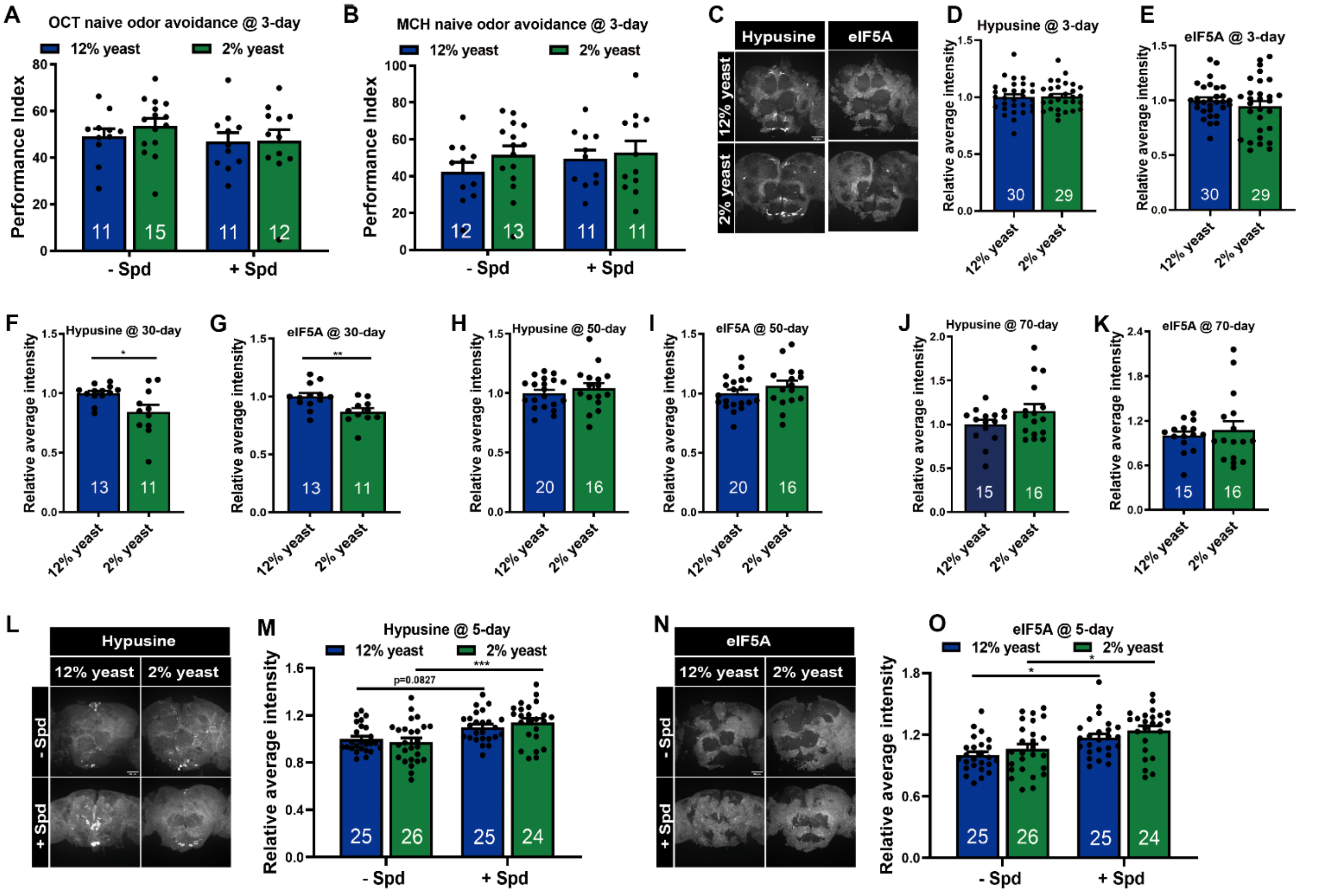


**Supplementary Figure 2. Naïve odor avoidance of OCT and MCH in young *w*$^{1118}$ flies in different diets and protein restriction does not boost eIF5A hypusination.** (**A**) Naïve odor avoidance of 3-Oct in young isogenic *w*$^{1118}$ flies (n = 11 – 15 biological replicates. Each biological replicate contains around 70 flies). (**B**) Naïve odor avoidance of MCH in young isogenic *w*$^{1118}$ flies (n = 11 – 13 biological replicates. Each biological replicate contains around 70 flies). (**C**) Confocal images of hypusine in 3-day-old *w*$^{1118}$ fly brains, bred in 12% yeast and 2% yeast. (**D**) Quantification of confocal brain staining for average hypusine intensity of 3-day-old *w*$^{1118}$ flies, bred in 12% yeast and 2% yeast (n = 29 – 30 fly brains). (**E**) Quantification of confocal brain staining for average eIF5A intensity of 3-day-old *w*$^{1118}$ flies, bred in 12% yeast and 2% yeast (n = 29 – 30 fly brains). (**F**) Quantification of confocal brain staining for average hypusine intensity of 30-day-old *w*$^{1118}$ flies, bred on either 12% yeast or 2% yeast (n = 11 – 13 fly brains). (**G**) Quantification of confocal brain staining for average eIF5A intensity of 30-day-old *w*$^{1118}$ flies, bred on either 12% yeast or 2% yeast (n = 11 – 13 fly brains). (**H**) Quantification of confocal brain staining for average hypusine intensity of 50-day-old *w*$^{1118}$ flies, bred on either 12% yeast or 2% yeast (n = 16 – 20 fly brains). (**I**) Quantification of confocal brain staining for average eIF5A intensity of 50-day-old *w*$^{1118}$ flies, bred on either 12% yeast or 2% yeast (n = 16 – 20 fly brains). (**J**) Quantification of confocal brain staining for average hypusine intensity of 70-day-old *w*$^{1118}$ flies, bred on either 12% yeast or 2% yeast (n = 15 – 16 fly brains). (**K**) Quantification of confocal brain staining for average eIF5A intensity of 70-day-old *w*$^{1118}$ flies, bred on either 12% yeast or 2% yeast (n = 15 – 16 fly brains). (**L**) Confocal images of hypusine in 5-day-old *w*$^{1118}$ fly brains, bred in 12% yeast and 2% yeast with and without addition of spermidine (n = 24 – 26 fly brains). (**M**) Quantification of confocal brain staining for average hypusine intensity of 5-day-old *w*$^{1118}$ flies, bred on either 12% yeast or 2% yeast with and without addition of spermidine (n = 24 – 26 fly brains). (**N**) Confocal images of eIF5A in 5-day-old *w*$^{1118}$ fly brains, bred in 12% yeast and 2% yeast with and without addition of spermidine (n = 24 – 26 fly brains). (**O**) Quantification of confocal brain staining for average eIF5A intensity of 5-day-old *w*$^{1118}$ flies, bred on either 12% yeast or 2% yeast with and without addition of spermidine (n = 24 – 26 fly brains). *p < 0.05, **p < 0.01, ***p < 0.001, ****p< 0.0001, ns, not significant. Data are mean ± SEM. P-values were determined by unpaired two-tailed t-test (**D**, **E**, **F**, **G**, **H**, **I**, **J**, **K**) and 2-way ANOVA with Tukey's post hoc multiple comparisons test (**A**, **B**, **M**, **O**).
